# Loudness changes as a function of the audiovisual scene

**DOI:** 10.1038/s41598-026-58091-z

**Published:** 2026-06-25

**Authors:** Liesbeth Gijbels, Kaylah Lalonde, Yi Shen, Mark T. Wallace, Adrian KC Lee

**Affiliations:** 1 Department of Speech & Hearing Sciences, University of Washington, Seattle, WA USA; 2 Institute for Learning & Brain Sciences, University of Washington, Seattle, WA USA; 3 Center for Hearing Research, Boys Town National Research Hospital, Omaha, NE USA; 4 Department of Psychology, Vanderbilt University, Nashville, TN USA

**Keywords:** Neuroscience, Psychology, Psychology

## Abstract

Understanding how listeners perceive speech in complex audiovisual (AV) environments is essential for characterizing communication in everyday settings. Here, we used loudness ratings to examine how AV scene characteristics shape the perceptual representation of a target talker in multitalker babble. Twenty-five normal-hearing adults rated the perceived loudness of a female talker presented in four-talker babble at four target-to-masker ratios (TMRs: − 21, − 9, − 6, − 3 dB). Audiovisual temporal coherence was manipulated by delaying the audio relative to the video at five stimulus onset asynchronies (SOAs: 0–500 ms), and three linguistic categories were tested: words, pseudowords, and reversed words. At synchronous presentations, loudness ratings scaled with TMR and were higher for words than pseudowords and reversed words, despite identical physical intensities. Increasing AV asynchrony reduced perceived loudness, with significant drops emerging beyond 150–300 ms SOA. Mapping loudness ratings onto perceived TMR change using individual loudness-growth slopes eliminated the main effect of the linguistic category, isolating a 2–3 dB perceived TMR drop at 500 ms SOA across all categories. These results demonstrate that both AV temporal synchrony and linguistic content shape loudness perception in noise, but their contributions are dissociable. This provides a novel view into how the perceptual construct of loudness changes with the AV scene, with implications for how loudness ratings can be used to study scene analysis and communication.

## Introduction

Conversational contexts are one of the most prevalent and relevant social experiences, yet can be rather challenging in everyday settings. Traditional measures of listeners’ success in such settings amidst different competing masking sounds are focused on speech intelligibility (e.g.^1,2^). However, these studies inevitably face challenges in isolating factors associated with acoustic, linguistic, and cognitive demands of the task, as well as with individual differences in listeners’ abilities and effort^3,4^. This is particularly challenging when studying populations with diverse communication abilities, which can include minimally or non-verbal individuals. Ultimately, it is important to develop experimental protocols that can dissociate the contributions of prelinguistic processing from those of linguistic content in AV scene analysis.

Coherent visual signals provide potent temporal cues to improve AV scene analysis. It has been well established that extracting visual information from the target talker’s face improves speech intelligibility^2^, particularly in noisy environments (i.e., adverse target-masker ratios [TMR]^1^). Visual (i.e., articulator movements) and auditory (i.e., acoustic envelope) information of AV speech are inextricably correlated due to the physical constraints related to speech production, with visual cues preceding auditory cues in natural AV speech (viz., 100–300 ms^5^). To deal with such offsets, our perceptual system can accommodate a range of AV asynchronies and still perceive the information as originating from the same object or event^6^. This range of asynchronies is captured by the construct known as the Temporal Binding Window (TBW), within which there is a high probability of the signals being perceptually bound. Much of the work on the TBW has focused on subjective timing (i.e., simultaneity) judgements and how stimulus characteristics influence these judgements (e.g.^7^). In contrast, little work has focused on the perceptual characteristics of the bound object.

Here we asked listeners to judge how loud the target talker (i.e., video of female talker) is perceived in the presence of a four-talker female babble masker as a proxy on how well they selectively attend the target. Four TMR levels were used: one to establish a control level (TMR = −21 dB; the floor of the loudness range), and three with a set TMR increment (−9, −6, −3 dB). These levels allowed us to map individual subjective loudness ratings to objective perceived TMR dB measures. During presentation of the target talker video, a lag of the audio signal was introduced at 5 stimulus onset asynchronies ([SOAs] of 0, 50, 150, 300, 500 ms) with the visual stimulus always leading. This manipulation allows us to ask how changing the AV temporal structure alters the loudness of the perceived target. Speech stimuli of different linguistic categories: words, pseudowords and reversed words were used to examine how linguistic information could influence loudness perception and AV scene analysis.

We hypothesized AV asynchrony could affect perceived loudness, as coherent AV presentation typically facilitates speech processing^8,9^ and as temporal certainty improves auditory detection^10^. TMR was expected to have a robust effect on loudness ratings, serving as a control for the reliability of the task. For the linguistic manipulation, we hypothesized that words would be rated as louder than pseudowords and reversed words, given that linguistic accessibility may facilitate target segregation from the masker, thereby enhancing perceived loudness.

## Methods

### Ethics statement and recruitment

Participants were recruited either through the University of Washington or via oral and online advertisements. Recruitment materials included a flyer distributed to undergraduate and graduate students in the Speech and Hearing Sciences and Engineering departments at the University of Washington, as well as advertisements posted on social media to reach a broader demographic. A dedicated website was created to inform prospective participants about the study, provide a platform for questions, and outline the steps for participating remotely. All participants gave informed consent remotely prior to each task, in accordance with a protocol approved by the University of Washington’s Institutional Review Board (STUDY00003064); all methods were performed in accordance with the relevant guidelines and regulations. The experiment would not proceed without confirmed consent. Participants received compensation for their time at a rate of $15 per hour, delivered via an online gift card.

This work was part of a larger body of work, answering four different research questions, in four separate experiments (Gijbels et al., 2024). All experiments were implemented remotely and any participant that completed experiment 2, 3, or 4, had already completed experiment 1. The experiment presented here was experiment 3. Experiment 1 and 2 investigated synchrony perception of AV speech, and its reliability^11^.

### Participants

Participants were eligible for inclusion based on the following criteria: (1) being between 18 and 40 years of age, (2) residing in the United States (to facilitate participant compensation), (3) identifying as a native speaker of English, (4) self-reporting normal hearing thresholds, and (5) self-reporting normal or corrected-to-normal vision. These criteria were assessed through a self-report questionnaire.

To confirm normal hearing ability, participants completed a brief sentence recognition task in quiet at the beginning of the first session. Only those who achieved a perfect score were allowed to continue in the study. The task included six trials randomly selected from a set of ten sentences spoken by a female native English speaker^12^. As described in this original study and relevant for this task, these sentences were syntactically correct but semantically neutral (low in predictability). For each trial, participants listened to an auditory stimulus and had to type the sentence in a text box. No feedback was provided. Questions about age, race, and Hispanic ethnicity were modeled after items from the 2020 U.S. Census.

Forty-six participants from task one^11^ were invited to take part in this remote loudness perception task. Among them, 27 participants consented to participate. The average age of the participants was 28.3 years (median = 27, SD = 5.91, min = 21, max = 40), with 4 identifying as male and 23 as female. All participants completed the task within two months of the initial task.

### Experimental setup and task

The tasks were completed remotely but offline. Concerns regarding exact stimulus presentation for these online assessments of temporally sensitive measures like the TBW were addressed by executing the experiment remotely but offline and pre-assembling all stimuli. Participants received instructions via email and through a dedicated website. All tasks were carried out in the participants’ homes on their personal computers. Similar remote study designs have yielded reliable and reproducible results, even for children, whose attentiveness is more difficult to manage^13,14^. The tasks for all phases were created using the free online tool, Lab.js^15^.

Participants were instructed to complete the tasks in a quiet setting, using a computer (rather than a tablet or phone) with built-in speakers. They were asked not to wear headphones so that the auditory and visual signals were roughly co-located in both azimuth and distance, with the built-in speakers positioned near the visual display, thereby providing a more naturalistic free-field listening experience. They were asked to use either Google Chrome or Firefox browsers to complete the tasks offline. At the start of the first task, participants were asked to adjust the speaker volume to a comfortable level using a repeated English speech sample. They were instructed not to alter the volume during the entire experiment and to report the volume setting (on a scale from 0 to 100%) to ensure consistent intensity across all tasks.

### Experimental paradigm

**Fig. 1:**
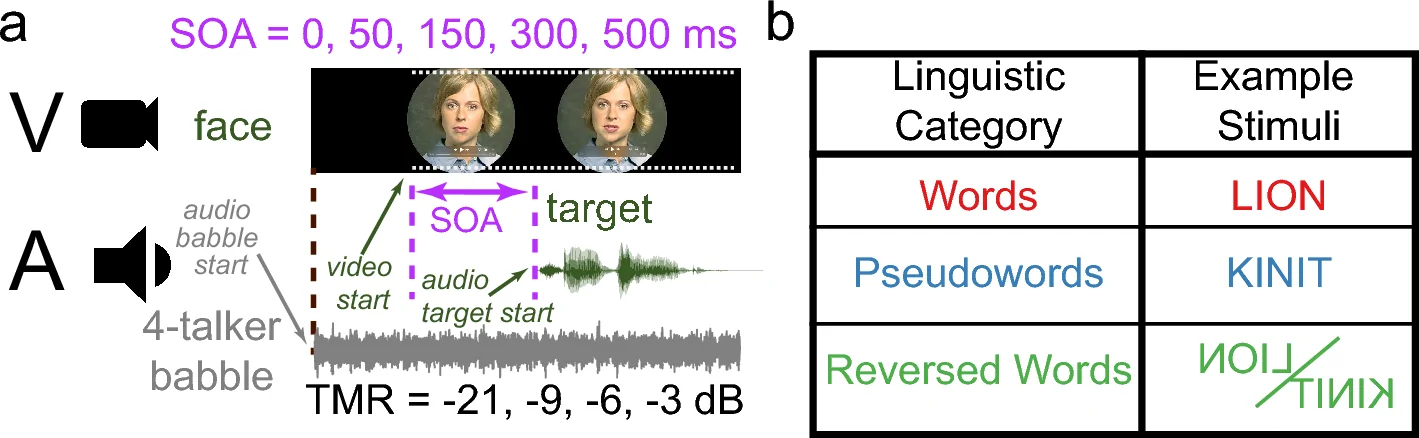
(**a**) AV paradigm and (**b**) Linguistic category and example stimuli. The photograph in panel (a) is reproduced with permission of the copyright holder, Rachel Holt, and is published under a CC BY 4.0 license.

Figure 1a shows the AV paradigm. Across all experiments, the stimulus onset asynchrony (SOA) was varied between 0 and 500 ms. The video was either presented simultaneously with or prior to the corresponding audio. Only visual-leading asynchronies were used for three reasons: (1) audio-leading events are uncommon in natural speech contexts^5^, (2) auditory-leading stimuli are not typically correlated with audiovisual speech perception scores^7,16^, and (3) limiting the SOA range helped keep each task within a manageable time frame for single-session completion. The choice of SOAs within this range was informed by earlier studies using speech stimuli^7^, which demonstrated that this window captures the full range of synchrony-asynchrony perception for speech. We opted for a randomized presentation of fixed SOA intervals (0, 50, 150, 300, and 500 ms) instead of using an adaptive staircase method, as prior findings^17^ suggest that preceding stimuli in adaptive procedures can bias synchrony judgments. All SOAs were created offline using ffmpeg via Python 3.7 to precisely align the auditory tracks with the corresponding silent video clips.

#### Stimuli, noise fragments and AV stimuli

The target stimuli for this experiment, training, and screening were sourced stimuli that have been validated in previous work^12,18–25^ and sentences from the Speech Test Video Corpus (SteVi^26^). For each linguistic level, three different female speakers were selected, with a total of 15 different stimuli per linguistic level. The word, pseudoword, and reversed speech stimuli were selected such that each category contained an equal amount of stimuli with 1, 2, or 3 syllables. We introduced word stimuli with different syllable lengths to limit the effect of absolute duration and envelope information. The reversed speech was a selection of both word and pseudoword stimuli.

As described in a related study by Gijbels et al.^11^, each stimulus was presented in a background of noise, specifically a four-talker babble masker (mean F0 = 196.5 Hz), chosen to provide a combination of energetic and informational masking, as used by Van Engen and colleagues (2014). The target speech (mean F0 = 169.6–212.9 Hz) occupied a similar fundamental frequency range as the masker. Furthermore, a four-talker babble was chosen to balance between maximizing informational masking while minimizing the potential of glimpsing due to momentary decreases in masker energy^27^. Each masker segment was a randomly selected 4000 ms excerpt featuring four female American English speakers producing meaningful sentences^28^. The four-talker babble was normalized for root mean square (RMS) amplitude using Audacity 2.4.2. The target stimulus began playing at a random interval between 500 and 1000 ms after the onset of the masker, with a target-to-masker ratio (TMR) of −21, −9, −6, or −3 dB. All target and masker files were pre-combined and processed offline.

In order to obtain a wider range of loudness ratings, we introduced variability in the intensity of the stimuli by utilizing three different TMR levels. Consequently, all stimuli (15 per linguistic category) were presented at each of the 5 SOAs at 3 different TMRs (−3, −6, and −9 dB). The intensity of the babble masker was kept constant across all TMR conditions, while the target speaker’s intensity was varied to achieve the specified TMRs (i.e., the target was added at varying intensities relative to the fixed-level masker). These TMRs, set in increments of 3 dB, were structured so that we have an objective benchmark of subjective loudness rating with objective TMR changes. Additionally, we included 15 trials at TMR = −21 dB and SOA = 0 ms, for each linguistic category. This control condition served as a reference loudness estimate, since a −21 dB TMR should be close to undetectable for all participants.

Video was compiled similarly as previously described in a related study (Gijbels et al., 2024 & 2025). Specifically, iMovie was used to select the size of the video frame so all speakers were equally visible (head and shoulders filling 90% of height of the frame: 1280 × 720 px), to cut the length of the videos to 2500 ms, to split the audio from the video fragment, and to place a black frame around the video, thus making all videos more similar. The speaker appeared in a cut-out circle. Before and after the 2.5 s-fragment black frames were added to make all videos exactly 4000 ms long.

ffmpeg software (Python 3.7) was used to select random masker fragments of 4000 ms, assemble target and masker with the specified TMR, and to combine audio and video (with the selected SOAs).

#### Linguistic levels

All speech stimuli were recorded with matched audio and video, ensuring natural alignment between the speaker’s voice and facial movements. No dubbing or artificial alterations were applied, except for the controlled introduction of temporal asynchrony by delaying the audio. The experimental design included three linguistic categories (Fig. 1b). At the lowest level, intended to assess AV correspondence without lexical processing, participants were presented with reversed speech—either real words or pseudowords played backwards. This condition, referred to collectively as reversed audiovisual speech, preserves the overall temporal envelope, including onset and offset cues, amplitude fluctuations, and cross-modal phase relationships. However, reversing the audio disrupts the typical phoneme-viseme associations found in American English^29^. Because of this, reversed speech may resemble the perceptual experience of listening to an unfamiliar language^30,31^. We argue that reversed speech more reliably severs phoneme-viseme associations, unlike foreign languages, which may still share some visemic characteristics with the listener’s native language^32^.

For the intermediate linguistic category, we used pseudowords (e.g., KINIT) — non-lexical items that conform to the phonotactic rules and syllable structure of English, while offering the same visemic and phonemic properties and durations as real words. Since pseudowords carry no meaning, factors such as word frequency and neighborhood density are not relevant. The highest linguistic level included real English words.

#### Loudness measure

Altogether, the task consisted of 720 trials, organized into 9 blocks, each containing 80 trials. These blocks were evenly distributed across the linguistic categories, with 3 blocks per level. The TMRs and SOAs were randomly distributed within these blocks. Additionally, the sequence of these 9 blocks was randomly determined.

Each trial consisted of the presentation of a fixation cross (500 ms), followed by an AV stimulus presentation (4000 ms) and a participant response (c.f.^11^). Under the video presentation the participant was shown the question “How loud did you perceive the talker of the video?” The participant was asked to use a slider, positioned beneath the question, to assess the loudness of the talker in the video on a scale ranging from 0 to 100 (with unit increments). Using this method, we were able to obtain 15 loudness ratings (between 0 and 100) per TMR, SOA, and linguistic category.

Participants were instructed to adjust the intensity of their computers to match the percentage indicated in the first experiment of this experiment series (i.e., the synchrony perception task described in^11^), in which they had set their speakers to a comfortable loudness level based on a repeating English speech fragment and provided an estimate of this setting (0–100%). However, determining loudness ratings, particularly in noisy environments, is highly subjective^33^. To ensure that participants understood the expected range of loudness ratings, we implemented a training phase. During the training, participants were first presented with a sentence at a TMR of −3 dB (SOA = 0 ms) and were informed that this represented the maximum loudness level of the task. Accordingly, for such stimuli, we provided feedback that trials like these should be rated in the upper quartile of the slider (75–100%). Subsequently, participants were presented with a stimulus at a TMR of −21 dB (SOA = 0 ms), signifying the quietest level of the task. Participants were instructed that stimuli like this should be rated between 0–25%. These examples were followed by 12 practice trials, where participants were randomly presented with words, pseudowords or reversed words per TMR (−3, −6, −9, −21 dB) and were asked to use the slider to rate the loudness of the talker in the video. Following each video, feedback was provided regarding the expected loudness range for their response in quartiles (0–25%, 25–50%, 50–75%, 75–100%).

### Data analysis

Statistical analyses were performed using packages from the tidyverse^34^, along with nlme^35^, emmeans^36^, and gamm4^37^ in R and RStudio (version 4.5).

#### Outliers

To define outliers for this task we calculated z-scores based on the mean and standard deviation of the loudness scores, per linguistic category, SOA and TMR. Shapiro–Wilk tests on participant-averaged loudness scores confirmed that normality was not violated in 43 of 45 condition cells (96%; all p > 0.05), with only two marginal exceptions (p = 0.045 and p = 0.031). Skewness and kurtosis values remained within acceptable limits across all cells (|skewness|< 1.1, |kurtosis|< 1.3). We then collected a summary of the outlier conditions (|z-score|> 2) for each participant^38^. Individuals with more than 5 outlier scores would be considered general outliers and removed from the dataframe. Two out of 27 participants were excluded based on these criteria.

#### Loudness rating by linguistic category

We used a linear mixed effects model (Model 1: Loudness rating ~ linguistic category * TMR + (linguistic category + TMR|participant)) for synchronous AV stimuli (SOA = 0 ms), to interpret differences in loudness ratings for the three different linguistic categories and 4 TMRs.

For the random effects, participant was included as a random intercept and linguistic category and TMR as random slopes.

#### Loudness rating by temporal asynchrony (and linguistic category)

We used a generalized additive model (Model 2: Loudness rating ~ SOA*TMR *linguistic category + (TMR + linguistic category + SOA|participant)), to interpret the effects of different SOAs on the loudness rating, for each linguistic category (words, pseudowords, reversed words) and each TMR (−9, −6, and −3 dB). Participant was included as a random intercept and TMR, linguistic category and SOA as random slopes. This generalized additive model was chosen to handle the non-linearity of the loudness rating in function of SOA, with no need to predefine the shape.

#### Mapping of perceived loudness to perceived TMR

By omitting our reference TMR (−21 dB), we retained 3 TMRs with equal steps (3 dB). To map our subjective perceptual measure (i.e., loudness rating in %), into an objectively interpretable score (TMR change in dB), we first created individual slopes per participant that represented loudness ratings for synchronous AV stimuli (SOA = 0 ms) by TMR, per linguistic category. The stepwise increase in loudness rating for each step of the TMR-scale (i.e., slope), then provided us with a mapping (per individual and as a group) for what percentage of perceived loudness matched with 1 dB in TMR change for each individual.

These individual slopes come from a linear regression of the loudness rating for synchronous AV presentation by TMR (−9, −6, −3 dB), per participant and linguistic level. This assumes a linear relationship between loudness rating and TMR. If that relationship is non-linear the TMR mapping could be distorted. When calculating the coefficient of determination (R2) from a linear fit between −9 and −3 dB TMR we observe that the linear model accounts for over 90% of the variance in loudness ratings for 79% of participant × linguistic level combinations, suggesting the linear approximation is reasonable for most participants. Seven fits fall below R2 < 0.85, all in the reversed words condition, indicating that the loudness-to-TMR mapping should be interpreted with slightly more caution for the reversed word category.

Second, we calculated the change in perceived loudness caused by asynchrony for each individual by subtracting the loudness rating at each asynchronous SOA from the synchronous rating (SOA = 0 ms), for each linguistic category and TMR: *ΔL(SOA)* = *L(SOA) − L(SOA* = *0)*. Dividing this by the individual’s slope (*β*) yields the perceived TMR drop in dB: *ΔTMR(SOA)* = *ΔL(SOA)/β*. This allowed us to map, for each individual and on a group level, what the perceived TMR drop (dB) was for each introduced SOA.

We reran Model 2, but changed loudness rating into the perceived TMR change (dB): Model 3 = Perceived TMR change ~ SOA*TMR *linguistic category + (TMR + linguistic category + SOA|participant).

## Results

**Fig. 2:**
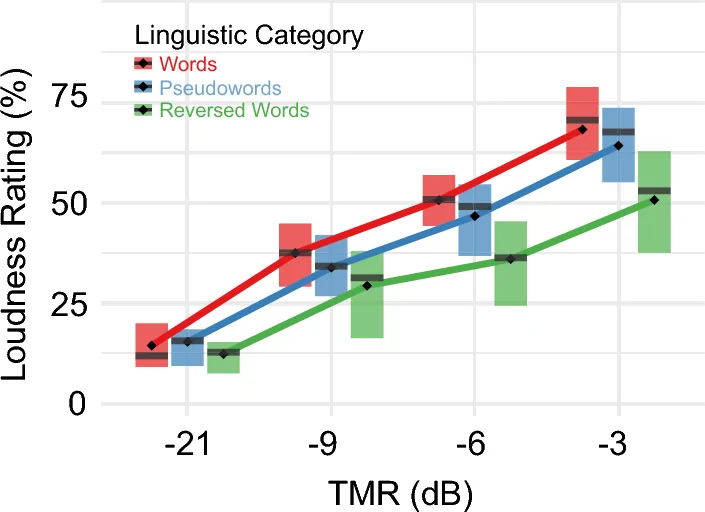
Loudness rating for each linguistic category at SOA = 0 ms. Vertical shaded areas denote interquartile range with horizontal bars denoting their medians (diamonds denote their means).

First, we considered the loudness ratings of the target talker reported by the participants for synchronous (SOA = 0 ms) AV stimulus presentations only (Fig. 2). Across all three linguistic categories, loudness ratings increased significantly with TMR (F_1, 4167_ = 272.42, p < 0.0001; b = 2.79, SE = 0.17, t = 16.92, p < 0.001), following the expected pattern for the TMRs tested (i.e., − 21 < − 9 < − 6 < − 3 dB), confirming that listeners can use the loudness rating scale reliably. Across TMRs, loudness ratings differed significantly between linguistic categories (F_2, 4167_ = 18.57, p < 0.0001), despite their identical physical intensities. Relative to words, both reversed words (b = − 11.09, SE = 1.92, t = − 5.78, p < 0.001) and pseudowords (b = − 2.80, SE = 1.06, t = − 2.63, p = 0.009) were rated substantially lower, suggesting that linguistic information directly affects how loud the target talker is perceived. There was also a significant interaction between TMR and linguistic category (F_2, 4167_ = 27.29, p < 0.0001): the loudness growth rate per dB TMR was shallower for both reversed words (b = − 0.81, SE = 0.11, t = − 7.30, p < 0.001) and for pseudowords (b = − 0.28, SE = 0.11, t = − 2.59, p = 0.010) compared to words.

**Fig. 3:**
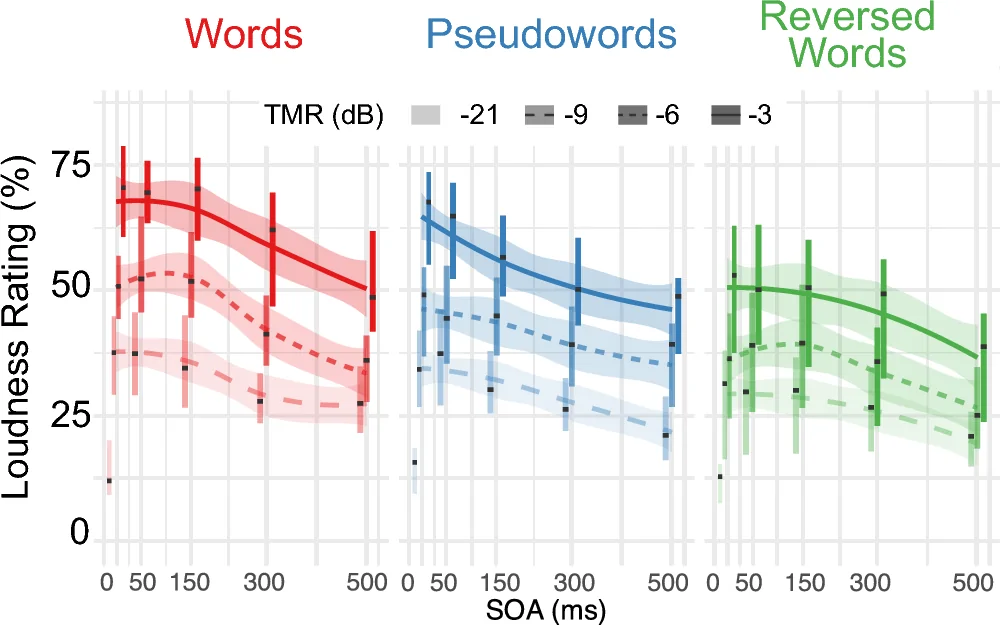
Loudness rating for each linguistic category across different SOAs. Vertical shaded areas denote interquartile range with horizontal bars denoting their medians (diamonds denote their means). Non-parametric least-mean-square fitting across SOAs (and the associated standard error in shaded areas).

Next, we analyzed loudness perception as a function of temporal asynchrony (i.e., SOA; Fig. 3). Across linguistic categories and TMRs, we observed that the loudness ratings dropped as SOAs grew larger (F_1,12_ = 102.42, p < 0.0001). Furthermore, the main effects for TMR (F_1,12_ = 335.82, p < 0.0001, b = 4.81, SE = 1.55, t = 3.09, p = 0.002) and linguistic categories (F_2,12_ = 27.10, p < 0.0001) remained present across SOAs, with reversed words rated significantly lower than both words (b = − 11.59, SE = 2.37, t = − 4.88, p < 0.001) and pseudowords (b = − 6.84, SE = 2.45, t = − 2.80, p = 0.005).

All 2-way interactions were significant: SOA x TMR (F_1, 12_ = 13.50, p < 0.0001; b = − 0.002, SE = 0.001, t = − 3.69, p < 0.001), indicating a steeper loudness decline at higher TMRs; SOA x linguistic category (F_2, 12_ = 10.56, p < 0.0001), where the SOA-related loudness decline was attenuated for reversed words (b = 0.010, SE = 0.002, t = 4.56, p < 0.001) and pseudowords (b = 0.004, SE = 0.002, t = 1.99, p = 0.046) relative to words; and TMR x linguistic category (F_2, 12_ = 45.05, p < 0.0001), where the TMR slope was shallower for reversed words (b = − 1.44, SE = 0.15, t = − 9.30, p < 0.001) and pseudowords (b = − 0.46, SE = 0.15, t = − 3.00, p = 0.003) compared to words. No three-way interaction was observed (p > 0.31).

These interactions indicate that the effect of SOA on loudness ratings was modulated by both TMR and linguistic category: the decline in loudness with increasing SOA was steeper at more favorable TMRs, and linguistic categories showed differential sensitivity to temporal asynchrony. Similarly, the effect of TMR on loudness differed across linguistic categories, with reversed words showing a shallower loudness growth rate.

**Fig. 4:**
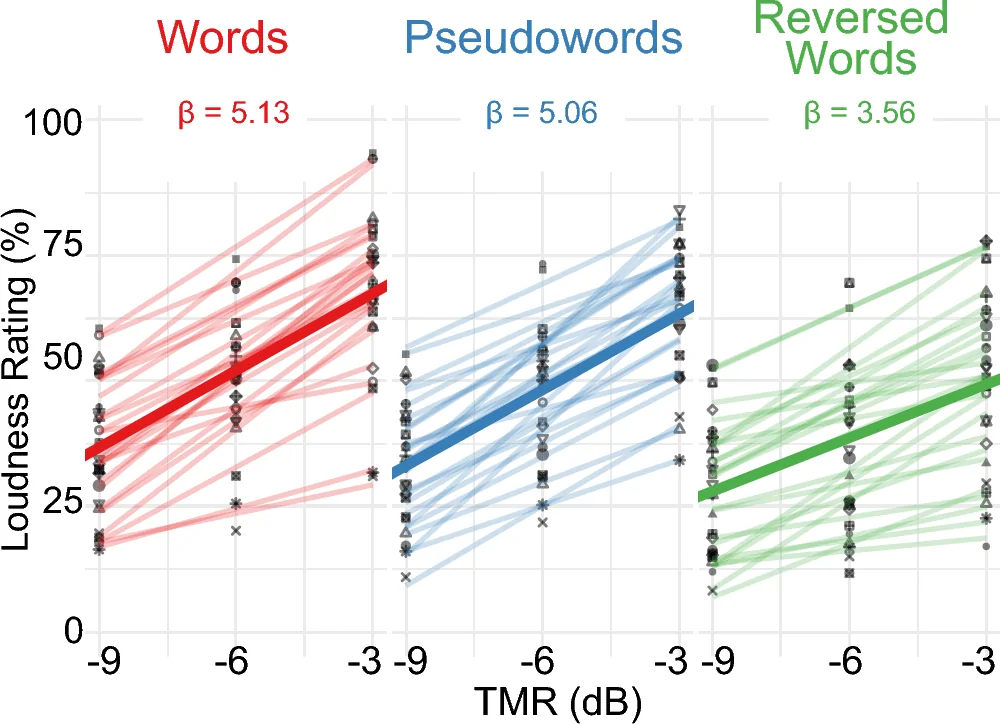
Loudness rating for each linguistic category as a function of TMR.

**Fig. 5:**
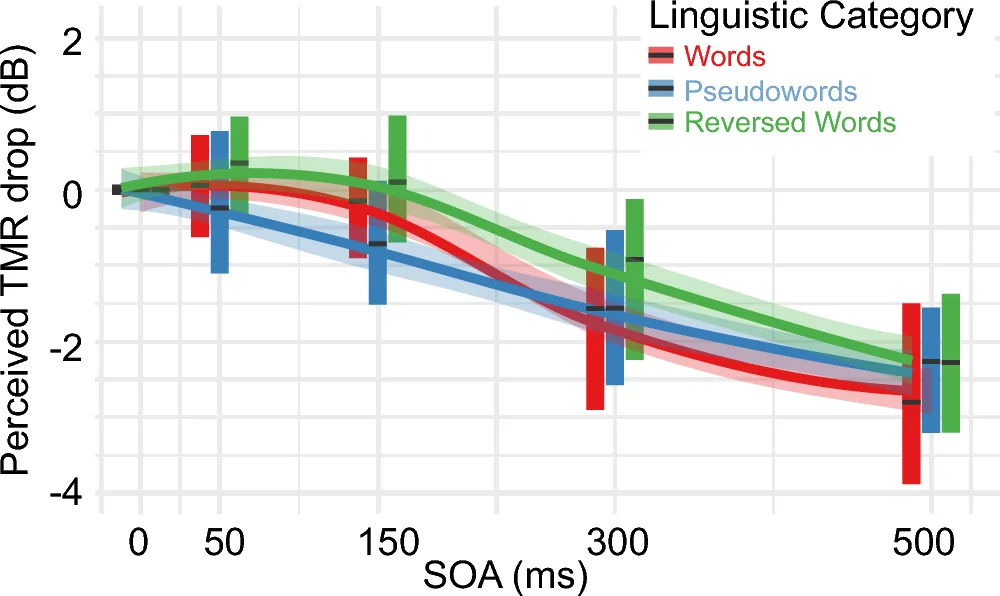
Mapped perceived TMR drop metric for each linguistic category. Vertical shaded areas denote interquartile range with horizontal bars denoting their medians. Non-parametric least-mean-square fitting across SOAs (and the associated standard error in shaded areas).

Plotting the slope of the loudness rating growth at synchronous AV presentations for each TMR reveals that the growth of loudness for reversed words is the least compared to the other two categories (Fig. 4). The mean loudness growth rate was β = 5.13/dB for words and β = 5.06/dB for pseudowords, compared to β = 3.56/dB for reversed words. Pairwise comparisons of TMR slopes were obtained using estimated marginal trends (emtrends in emmeans) from the linear mixed-effects model. Both words (t_48_ = 3.23, p = 0.002) and pseudowords (t_48_ = 3.07, p = 0.003) had significantly steeper slopes than reversed words, while words and pseudowords did not differ from each other (t_48_ = 0.19, p = 0.851). We then used the individual’s slope to map the asynchronous loudness rating, normalized by the synchronous condition, to a perceived TMR drop for each linguistic category at each SOA (Fig. 5). This mapping eliminated the main effects of linguistic level (b = − 0.64, SE = 0.88, t = − 0.73, p = 0.465 for pseudowords, and b = 0.24, SE = 0.88, t = 0.27, p = 0.789 for reversed words compared to words) and TMR (b = 0.01, SE = 0.10, t = 0.11, p = 0.911), unlike in Fig. 2 and 3. Now, the only remaining significant effect is that of SOA on the perceived TMR drop (F_1, 12_ = 28.11, p < 0.0001). Pairwise comparisons were conducted using paired t-tests comparing each SOA (50, 150, 300, 500 ms) to the synchronous condition (SOA = 0 ms), separately for each linguistic category (12 comparisons total, Holm-Bonferroni corrected). These reveal that the difference emerges only beyond SOA of 50 ms for pseudowords (b = − 0.97 dB at 150 ms, t_24_ = − 3.21, p = 0.004, d = −0.64) and 150 ms for words (b = − 1.82 dB at 300 ms, t_24_ = − 6.32, p < 0.001, d = −1.26) and reversed words (b = − 1.50 dB at 300 ms, t_24_ = − 2.90, p = 0.008, d = −0.58). When examined relative to the synchronous condition (i.e., 0 ms SOA), the drop in perceived TMR reaches ~ 2–3 dB across all linguistic categories at 500 ms SOA (words: − 3.10 dB; pseudowords: − 3.13 dB; reversed words: − 3.74 dB).

The disappearance of the linguistic category effect after mapping loudness ratings into perceived TMR drop (Fig. 5) indicates that the influence of linguistic content on loudness ratings is accounted for by differences in loudness-growth rate across categories. Thus, the rate at which loudness increases with TMR is steeper for linguistically accessible stimuli. This steeper growth rate could reflect more efficient segregation of the target from the masker when linguistic cues are available, effectively amplifying each decibel of physical TMR advantage into a larger perceptual gain. Once this differential growth rate is factored out through the individual slope normalization, the three linguistic categories converge, and only the effect of AV temporal coherence remains.

A post-hoc power analysis was conducted to evaluate the sensitivity of the study (N = 25) for the reported effects. For the main within-subjects effects of TMR and linguistic level at SOA = 0 ms, observed effect sizes were large (partial η2 = 0.49–0.90, Cohen’s f = 0.98–2.94), yielding achieved power > 0.99 at α = 0.05. A sensitivity analysis further confirmed that with N = 25, the study could reliably detect effects as small as Cohen’s f = 0.37 (80% power), well below the observed values.

## Discussion

Although loudness is a well-studied perceptual construct, the vast majority of the work has focused on it as a function of physical intensity, as well as how it is influenced by frequency, the degree of hearing loss and binaural cues^39^, with cognitive factors largely unaccounted for^40^. In contrast, little is known about how linguistic category or AV scene analysis influences loudness perception with speech stimuli (except for^41^). Our findings that different linguistic conditions influence the loudness rating of synchronous AV stimuli was rather surprising. A previous study showed no difference in loudness ratings between Hindi and English auditory speech stimuli for native English speakers^42^. However, the discrepancy with our findings can be attributed to several key methodological differences. First, Fucci and colleagues presented speech in quiet at varying sensation levels (10–90 dB SL), whereas our stimuli were embedded in four-talker babble at fixed TMRs. The presence of a competing masker likely engages selective attention and scene analysis processes that may amplify the perceptual advantage of linguistically accessible speech. Second, they used magnitude-estimation scaling, an open-ended, ratio-level method where participants freely assign numbers, whereas we used a bounded loudness rating scale (0–100%) with anchored endpoints and training, which may be more sensitive to subtle perceptual differences. Third, their linguistic manipulation compared two natural languages (English vs. Hindi), which differ in phonotactics, prosody, and talker identity, whereas our design matched speakers, duration, and syllabic structure across forward and reversed forms, isolating linguistic content from acoustic confounds. Fourth, they used audio-only presentation via headphones, whereas our paradigm was AV, introducing cross-modal binding processes that may differentially benefit stimuli with intact phoneme-viseme correspondences.

In addition, we found that the temporal structure of the AV pairing (i.e., degree of asynchrony) had a significant effect on loudness ratings. Loudness perception as a function of multisensory temporal asynchrony has, to our knowledge, only been investigated in one other study, examining auditory-somatosensory interactions^43^. In that study, synchronous tactile vibrations enhanced both auditory detection and perceived loudness of simple tones presented in quiet, whereas asynchronous tactile stimuli had no effect relative to a no-touch baseline. This enhancement was strongest for the weakest sound intensities (up to 68% increase), consistent with the principle of inverse effectiveness. Our findings extend these results in several important ways. First, we demonstrate that the temporal rule of multisensory integration applies to AV speech in noise, a more complex and ecologically relevant setting. Second, whereas Gillmeister and Eimer used a binary synchronous/asynchronous manipulation (0 vs. +/- 200 ms), our parametric SOA design (0–500 ms) reveals a graded decline in perceived loudness, with the enhancement remaining intact up to 150–300 ms of AV asynchrony. This tolerance window aligns with the natural statistics of AV speech, where visual cues typically precede auditory cues by 100–300 ms^5^, suggesting that the perceptual system accommodates ecologically plausible asynchronies before loudness perception is degraded. Third, the ~ 2–3 dB perceived TMR drop we observed at 500 ms SOA provides a quantitative measure of the cost of temporal incoherence, complementing Gillmeister and Eimer’s finding that synchronous touch produced intensity enhancements equivalent to physical increases in sound level.

Our findings also offer important perspectives when considered alongside established models of loudness perception. Moore^40^ noted that loudness perception can be influenced by high-level and cross-modal factors not accounted for in any current model, and that in everyday listening, the auditory system has evolved to estimate properties of auditory objects or sound sources, rather than the purely physical signals. The effects of linguistic category and AV temporal coherence on loudness ratings as reported by this work demonstrate that listeners’ loudness judgments reflect properties of the perceived auditory object, shaped by multisensory binding and linguistic accessibility, rather than solely the acoustic energy at the ear.

When we mapped loudness ratings into perceived TMR drop using individual loudness-growth slopes, the linguistic category effect disappeared, while the SOA effect remained. This suggests that the linguistic influence on loudness ratings may be captured by, and thus attributable to, differences in the rate at which loudness grows with TMR across linguistic categories, rather than reflecting an independent perceptual mechanism. In contrast, the persistent effect of AV temporal incoherence after this normalization indicates that it affects loudness perception through a pathway not accounted for by the loudness-growth function alone. This dissociation is consistent with Moore’s^40^ suggestion that high-level and cross-modal factors shape loudness perception through mechanisms distinct from those captured by current signal-based models.

Our mapping from loudness ratings to perceived TMR drop reveals that extreme asynchronous AV mismatch is associated with a 2–3 dB drop, independent of linguistic categories and the original TMR. Although tested in a different context, Egan and colleagues (1961) showed that auditory detection with temporal certainty resulted in 2–3 dB of improvement in detection, when compared to conditions with temporal uncertainty. This is in general agreement with other studies on detection advantage using coherent audio-visual speech^8,9^. Thus, temporal synchrony both within and across different sensory modalities exerts similar enhancing effects on perceptual judgments. Future investigations should explore whether this phenomenon generalizes to other stimuli and populations.

Using different TMRs provides a vital control check to ensure participants can reliably perform the loudness rating task, while the resulting slope of the loudness growth function can potentially serve as a novel index for quantifying processing differences between linguistic categories (e.g., comparing the slopes for words, pseudowords, and reversed words as a measure of the linguistic contribution to loudness perception). In addition, the perceived TMR drop as a function of SOA provides a measure of the perceptual effect on AV scene analysis.

Finally, the ability to collect robust results across sessions and across different linguistic categories in a remote setting provides a potential platform to study AV scene analysis in a more accessible way. While future work should establish how these findings compare with those obtained under more controlled laboratory conditions, the results obtained from these tasks indexing various facets of AV speech integration in a home setting provide optimism for extending this work to neurodiverse listeners who have a broad range of linguistic abilities.

## Data Availability

The data and analysis script that support the findings of this study are available from the following repository: https://zenodo.org/records/17381213
